# Factors Contributing to Complications and Failures of Impacted Canines Undergoing Surgical Orthodontic Treatment: A Retrospective Cohort Study

**DOI:** 10.3390/jcm15041463

**Published:** 2026-02-13

**Authors:** Yifat Manor, Maayan Kaganovich, Mor Gamliel, Noa Sadan, Tom Shmuly

**Affiliations:** 1 School of Dental Medicine, Gray Faculty of Medical and Health Sciences, Tel Aviv University, 69978 Tel Aviv, Israel; maayankaganovich@gmail.com (M.K.); 3mor30@gmail.com (M.G.); sadan.noa@gmail.com (N.S.); tom.shmuly@gmail.com (T.S.); 2 Dental Department, Oral & Maxillofacial Unit, Shamir Medical Center, 70300 Beer Yaacov, Israel

**Keywords:** impacted maxillary canine, canine exposure, surgical orthodontic treatment, treatment failure, complications

## Abstract

**Objectives:** This study aims to assess the prevalence of complications and failures associated with impacted canine eruption in a specialized referral center, with the goal of identifying factors that contribute to these outcomes. **Methods:** This retrospective cohort study included cases of impacted canines treated at the School of Dental Medicine between 2010 and 2020. Clinical and radiographic data were collected and evaluated for failures and complications by two independent clinicians (MK, MG). In addition, specialists in oral and maxillofacial surgery and orthodontics (YM, TS, NS) independently assessed all complications and failures. **Results:** Among the 214 impacted maxillary canines included, 23 (10.7%) failed to erupt following initial surgical–orthodontic treatment and required re-intervention. Eruption difficulty was attributed to orthodontic factors in 43.5% of cases, surgical factors in 13.0%, and combined factors in the remainder. Following a second procedure, 15 canines erupted successfully, while 8 did not, resulting in an overall failure rate of 3.7%. Treatment failure was significantly associated with both anatomical and procedural factors. Canines with centrally positioned crowns exhibited a significantly higher failure rate than those with buccal or palatal positions (χ^2^ test, *p* = 0.025). Failure was also more common when the canine root apex was located in close proximity to a cortical plate. Lateral incisor root resorption was significantly associated with treatment complications (*p* = 0.030). In the multivariable logistic regression analysis, root resorption remained an independent predictor of treatment failure, increasing the odds of failure approximately fourfold (OR = 0.255, CI = 0.077–0.843, *p* = 0.025). Timing and surgical technique were also significantly associated with treatment outcome. Surgical exposure performed shortly after diagnosis was linked to an increased risk of treatment complications (*p* = 0.006). Closed surgical exposure demonstrated a significantly higher failure rate compared with open exposure (Pearson exact test, *p* = 0.009). Although open exposure was associated with a greater likelihood of successful eruption, it was also significantly associated with increased gingival morbidity (Fisher’s test, *p* = 0.030). **Conclusions:** Failure of impacted maxillary canine eruption following combined surgical–orthodontic treatment is uncommon but is significantly associated with distinct anatomical and procedural risk factors. Central crown position, cortical plate involvement, lateral incisor root resorption, early surgical exposure, and the use of closed exposure techniques all increase the likelihood of treatment failure and complications. Although open exposure enhances the probability of successful eruption, it may also negatively affect gingival outcomes, underscoring the need for individualized, multidisciplinary treatment planning.

## 1. Introduction

Impacted maxillary canines are among the most common eruption disturbances encountered in orthodontic and oral surgery practice, with a reported prevalence of 1% to 3%. Their management remains a significant clinical challenge, although advances in diagnostic imaging have markedly improved treatment planning and risk assessment. Clinical diagnosis typically aligns with radiographic findings and is based on delayed eruption, persistence of the deciduous canine beyond ages 14–15, and the absence of a palpable soft-tissue bulge indicating the position of the permanent canine. Over the past decade, developments in three-dimensional imaging and interdisciplinary treatment planning have enhanced diagnostic precision and therapeutic decision making [1,2]. Recent studies highlight the pivotal role of cone-beam computed tomography (CBCT) in assessing canine position, proximity to adjacent roots, and cortical plate involvement, thereby improving risk stratification and guiding treatment strategies [3,4].

Palatal canine impaction is typically attributed to disturbances in arch length that disrupt the proper positioning of the tooth bud relative to the maxillary sinus and nasal cavity. In approximately 85% of cases, the underlying etiology is excess space within the maxillary bone, allowing the permanent canine bud to migrate within the available space and become fixed in an unfavorable position for eruption [5]. Additional causes include delayed exfoliation of the primary canine, ankylosis, cystic or neoplastic pathology, cleft palate, and other idiopathic factors [6]. Impacted permanent canines are frequently associated with anomalies of the adjacent lateral incisors, such as peg-shaped or congenitally missing teeth. Among patients with peg-shaped lateral incisors, 12.1% present with an impacted canine [7].

Neglected impacted canines may remain asymptomatic or may lead to a range of complications, including periodontal disease, aesthetic concerns, disturbances in arch-length development, root resorption of adjacent teeth, recurrent infections, and cyst formation [6,7].

The pattern of impaction is determined according to the Yamamoto classification for impacted canines:

Type 1: Vertically impacted canines positioned nearly perpendicular to the occlusal plane between the lateral incisor and first premolar.

Type 2: Canine impacted with a mesial inclination relative to the occlusal plane.

Type 3: Impacted canines tilted distally in relation to the occlusal plane.

Type 4: Horizontally impacted canines, with the crown directed medially.

Type 5: Horizontally impacted canines, with the crown directed distallyside.

Type 6: The canine is inverted.

Type 7: Impaction occurring in the labio-lingual (palatal) and ectopic positions.

This classification is made according to panoramic X-ray and can be useful for guiding treatment decisions and approaches for impacted canines [8].

The primary objectives of the surgical procedure are to expose the impacted canine to enable subsequent orthodontic traction, to remove any pathological tissue, and to eliminate anatomical obstructions that impede eruption, thereby creating an appropriate eruption pathway. The incision should be designed to minimize tissue trauma while avoiding unnecessary exposure of the cemento-enamel junction and the cervical third of the canine root [9].

Two principal surgical approaches are used to expose impacted canines during orthodontic treatment: the open and closed techniques. The open technique provides direct access to the impacted tooth by removing the overlying soft tissue and bone. After a flap incision is made, the flap may be apically repositioned to leave the crown exposed for natural eruption, or a window may be created to allow direct visualization and guidance of the erupting tooth [9]. In contrast, the closed technique involves raising a flap and removing a limited portion of the cortical bone to expose the crown, followed by bonding an orthodontic attachment to facilitate controlled traction. The flap is then repositioned and sutured to cover the tooth, allowing eruption to occur beneath the soft tissue [9,10].

Complications and eruption failure may arise from several factors, including inaccurate localization of the impacted canine, improper orthodontic traction direction, ankylosis resulting from early surgical intervention, and damage to the cementum or periodontal attachment apparatus during surgery [11]. Additional pathological conditions—such as odontomas, cysts, or supernumerary teeth—may also contribute to treatment failure [12]. Furthermore, insufficient keratinized tissue may predispose to gingival damage during or after the surgical procedure [13].

This retrospective study aims to evaluate the prevalence of impacted canine eruption failures at the School of Dental Medicine, a center specializing in complex dental cases. The study further seeks to identify the underlying causes of these failures, drawing on institutional experience to develop strategies that mitigate risk, optimize individualized treatment planning, and improve the management of complications. Although several risk factors have been described in the literature, limited evidence exists regarding the relative contribution of surgical versus orthodontic etiologies to treatment failure, as well as the prognostic significance of three-dimensional root apex localization in relation to the cortical plates.

## 2. Materials and Methods

This retrospective study included all cases of impacted canines treated with combined surgical–orthodontic therapy at the School of Dental Medicine of a university between 2010 and 2020. All treated cases of impacted canines were analyzed. Treatment outcomes—including successful eruption, complications, and failures—were independently evaluated by five clinicians. A successful outcome was defined as eruption of the canine into the dental arch. Complications were defined as events requiring re-operation, loss of tooth vitality, detachment of the orthodontic attachment, formation of scar tissue, gingival recession, or other gingival lesions. Failures were defined as cases when the canine was extracted or remained impacted.

Data evaluation included clinical photographs from the preoperative, intraoperative, and postoperative stages, as well as radiographic imaging. Clinical outcomes were correlated with demographic, clinical, and radiographic variables. The study was approved by the institutional ethics committee (TAU-0002231-5). Informed consent was waived due to the retrospective nature of the study, in accordance with institutional guidelines.

### 2.1. Inclusion Criteria

Patients who underwent surgical and orthodontic treatment for impacted canines at the institution.

Complete clinical and radiographic data: Clinical photographs from all the stages of the treatment and radiography—CBCT and panoramic X-rays.

Follow-up maintained until the end of orthodontic treatment.

### 2.2. Exclusion Criteria

Cases with other impacted teeth.

Cases with incomplete clinical or radiographic data.

**Figure 1:**
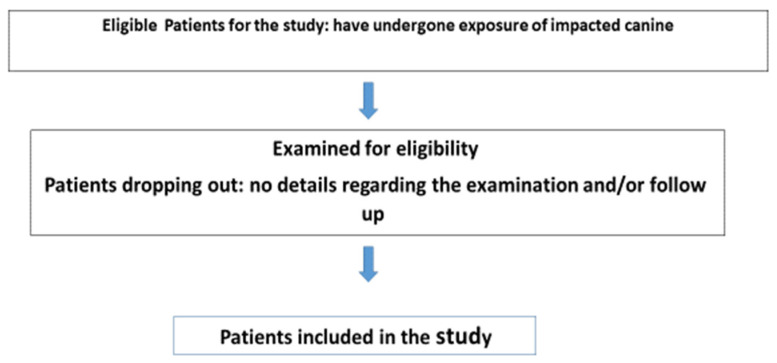
A flow chart of patients in the study.

A flow chart of patient selection is provided in Figure 1.

#### 2.2.1. Definitions and Outcome Measures

Treatment failure was defined as the inability of an impacted maxillary canine to erupt into the dental arch following both the initial and secondary surgical–orthodontic interventions, ultimately resulting in tooth extraction. Complications were defined as adverse outcomes occurring during treatment that did not necessarily lead to extraction; they included the need for a second surgical intervention followed by successful eruption, loss of tooth vitality, gingival scarring, gingival recession, root resorption, increased canine mobility and ankylosis. Ankylosis was diagnosed using combined clinical and radiographic criteria. During the second surgical procedure for canine exposure, ankylosis was further assessed according to the tooth’s lack of mobility in response to attempted luxation and by the presence of a dull sound on percussion.

#### 2.2.2. Etiology of Failures and Complications

Failures and complications were further categorized according to their primary etiology as surgical or orthodontic. Surgical-related failures were mainly associated with inappropriate choice of surgical approach (buccal instead of palatal), use of closed rather than open exposure, and insufficient removal of soft or hard tissue, which compromised the creation of an optimal eruption pathway. Surgical complications primarily involved soft tissue sequelae, including gingival scarring and gingival recession, and were related to traumatic flap handling, excessive soft tissue or bone removal, or impaired postoperative healing.

Orthodontic-related failures were predominantly attributed to incorrect traction vectors, inappropriate magnitude or direction of orthodontic forces, and appliance-related issues such as detachment of the bonded attachment from the impacted canine. Orthodontic complications included lateral incisor root resorption, gingival recession, increased canine mobility, and loss of tooth vitality. Cases where both surgical and orthodontic factors contributed to treatment failure were classified as combined etiology.

#### 2.2.3. Clinical and Radiographic Evaluation

Clinical photographs obtained before, during, and after treatment were independently evaluated by two clinicians (M.K., M.G.). Cases presenting eruption difficulties, the need for re-surgery or rebonding, loss of tooth vitality, periodontal complications, increased mobility, or extraction were subsequently reviewed by three additional clinicians (Y.M., T.S., N.S.). The underlying cause of each failure or complication was determined through consensus following joint discussion.

Recent evidence supports the use of three-dimensional CBCT imaging to classify impacted canine position and to predict adjacent root resorption with greater accuracy than panoramic radiography alone. Radiographic assessment in this study included panoramic radiographs, cone–beam computed tomography (CBCT), and intraoral radiographs. Impacted canines were classified according to the Yamamoto system using panoramic radiographs, independently evaluated by three clinicians (M.K., M.G., N.S.). CBCT scans were retrospectively reviewed to determine the crown position within the alveolar ridge, categorized as central or adjacent to a cortical plate (buccal or palatal).

#### 2.2.4. Inter-Rater Reliability Assessment

To assess the reliability of subjective clinical and radiographic evaluations, inter-rater agreement was calculated using Cohen’s kappa (κ) statistics. Agreement between the two clinicians involved in the initial screening of clinical photographs and eruption outcomes (M.K., M.G.) was evaluated using Cohen’s κ for categorical variables. For the assessment of causes of failure and complications, inter-rater reliability among the three clinicians (Y.M., T.S., N.S.) was evaluated using pairwise Cohen’s κ statistics, and the mean κ value was reported. Kappa values were interpreted according to Landis and Koch criteria (<0.20 poor, 0.21–0.40 fair, 0.41–0.60 moderate, 0.61–0.80 substantial, >0.80 almost perfect agreement). Ninety-five percent confidence intervals were calculated for all κ values. Discrepancies were resolved through consensus discussion only after independent assessments were completed.

Interpretation of the Kappa coefficient followed established benchmarks for clinical research reliability.

### 2.3. Statistical Analysis

The following variables were collected and analyzed:

Categorical variables: sex, side of impaction, Yamamoto classification, crown position (central vs. cortical), presence of lateral incisor root resorption (yes/no), surgical approach (open vs. closed), timing of exposure (early vs. delayed), occurrence of complications (yes/no), treatment outcome (success/failure).

Continuous variables: patient age at diagnosis and treatment duration.

Categorical variables were analyzed using the chi-square test or Fisher’s exact test, as appropriate. Continuous variables were assessed for normality using the Shapiro–Wilk test. Normally distributed data were analyzed using the independent-samples *t*-test, while non-normally distributed data were analyzed using the Mann–Whitney U test.

Binary logistic regression analysis was performed to identify independent predictors of treatment failure. The dependent variable was treatment outcome (failure = 1, success = 0). Independent variables entered into the model included crown position, lateral incisor root resorption, surgical exposure technique, and timing of exposure. Variables were coded dichotomously. Odds ratios (ORs) with 95% confidence intervals (CIs) were calculated.

All statistical analyses were performed using SPSS software (version 29.0; IBM Corp., Armonk, NY, USA). Statistical significance was set at *p* < 0.05.

## 3. Results

Of the 227 impacted maxillary canines initially identified, 214 met the inclusion criteria and were included in the analysis. Following the first surgical–orthodontic intervention, 191 canines (89.3%) successfully erupted into the dental arch, while 23 (10.7%) failed to erupt. Of these 23 cases, 15 underwent a second surgical intervention, resulting in successful eruption in 14 cases and failure in 1 case. Seven canines were extracted due to unfavorable anatomical or clinical conditions, and one case was excluded from further analysis due to lack of patient compliance. Overall, eight canines failed to erupt and were ultimately extracted, corresponding to a final failure rate of 3.7%.

**Figure 2:**
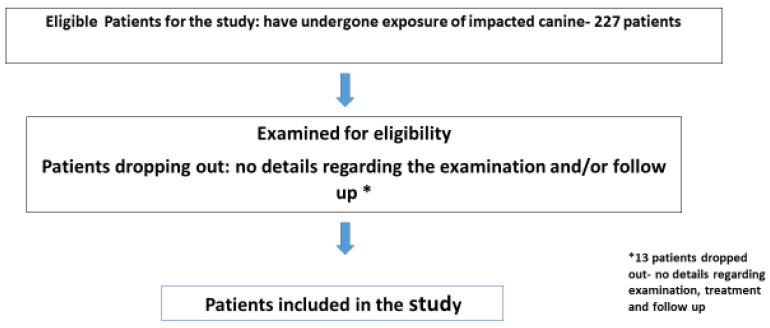
A flow chart of patients included in the study.

A flow diagram of patients included in the study is provided in Figure 2.

### 3.1. Descriptive Statistics

Of the 214 cases, 121 were female and 93 were male. The mean age at diagnosis was 14.6 years, and the mean age at the time of surgical exposure was 15.5 years. The average duration of treatment was 3.63 years. Case analysis is presented in Table 1, which shows complications and failures.

The clinical and radiographic findings are summarized in Table 2. The left maxillary canine was the most frequently impacted, with the palatal aspect representing the predominant impaction site. Bilateral impaction within the same jaw occurred in 45% of patients. Retained deciduous canines were present in 81.8% of cases. Most patients (93%) exhibited normal lateral incisor morphology, while 5.1% had peg-shaped incisors and 1.9% presented with microdontia. Root resorption of adjacent lateral incisors was identified in 18 cases. According to the Yamamoto classification, most impacted canines were categorized as Class I or II. Associated pathologies were identified in 3.7% of cases (Table 2 and Table 3).

### 3.2. Surgical Approach

Among the 191 canines that successfully erupted, 66 were treated using an open exposure technique and 125 using a closed exposure technique. Across the entire cohort of 214 cases, 144 canines (67%) underwent closed exposure at the initial procedure, whereas 70 (33%) were treated with open exposure. In re-exposure procedures, the proportion of open exposure increased substantially, accounting for 69% of cases, while the remaining 31% were managed using a closed technique.

Canine crown location (CBCT analysis): According to the CBCT evaluation (Table 3), the highest eruption success rates were observed when the impacted canine crown was positioned adjacent to the buccal plate (87%) or palatal plate (91%). In contrast, centrally positioned crowns demonstrated a markedly lower success rate of 62%. In these centrally located cases, the failure rate reached 38%, approximately three times higher than in the other groups. Among the 23 cases that presented complications, apex location was further analyzed: 12 involved root proximity to a cortical plate, and 6 ultimately required extraction of the impacted canine.

Most failures and complications were attributed to orthodontic factors.

Table 4 summarizes the 18 recorded complications: 44.4% presented soft tissue scarring, 22.2% showed root resorption at the end of treatment, 11.1% exhibited loss of vitality, 11.1% presented mobility, and 11.1% demonstrated gingival recession.

### 3.3. Inter-Rater Agreement

The two evaluators (M.K., M.G.) demonstrated a high level of concordance in case analysis. Out of 214 analyzed cases, identical classifications were assigned in 210 cases. As the level of inter-rater agreement met the predefined threshold for acceptability, the corresponding statistical data are not presented.

Regarding the analysis of types of complications or failure, out of 41 cases, identical classifications were assigned in 36 cases, yielding an observed agreement of (P_o = 0.878). The expected agreement by chance, based on the marginal category distributions, was (P_e = 0.305). Using these values, Cohen’s Kappa coefficient was calculated as: [kappa = 0.82]. This value indicates almost perfect agreement between the evaluators, consistent with accepted interpretive guidelines and supporting the reliability of the classification process used in the study.

### 3.4. Risk Factors for Complications and Failures

The risk factors for complications and failures for impacted canines undergoing surgical orthodontics are divided into preoperative and intraoperative factors.

### 3.5. Risk Factors Related to Preoperative Factors

1. Time interval between diagnosis and treatment: A significant correlation was found between a shorter interval from diagnosis to the initial surgical procedure and higher rates of failure and complications (*t*-test, *p* = 0.006).

2. Canine location within the alveolar ridge (CBCT): A significant association was observed between central crown position and increased failure and complication rates. Canines located centrally within the alveolar ridge—between the buccal and palatal cortical plates—demonstrated a significantly higher risk of unsuccessful eruption (chi-square test, *p* = 0.025).

3. Root resorption of the adjacent tooth: Adjacent lateral incisor root resorption was significantly associated with treatment complications and failure (logistic regression: B = −1.367, *p* = 0.025; OR = 0.255, 95% CI: 0.077–0.843). Clinically, the presence of lateral incisor root resorption corresponded to an approximately fourfold increase in the risk of treatment failure (OR = 4.0).

4. Proximity of the impacted canine root to the cortical plate: A significant correlation was identified between root proximity to a cortical plate and the occurrence of failures or complications (Pearson chi-square test, *p* = 0.025). Among the 23 cases with complications or failure, 12 involved root contact or close proximity to a cortical plate, and 6 ultimately required the extraction of the impacted canine.

### 3.6. Risk Factors Related to Intraoperative Factors

1. A significant association was found between the surgical approach and treatment outcomes. Open exposure demonstrated a markedly higher success rate (98.5%) compared with closed exposure (84.7%), whereas closed exposure showed a higher failure rate (15.3%) relative to open exposure (1.5%). This difference was statistically significant (chi-square test, *p* = 0.009). The odds of successful canine eruption following open exposure were 11.616 times higher than with closed exposure (95% CI: 1.532–88.099).
2. A significant association was also identified between the open exposure technique and surgical complications, particularly gingival damage (chi-square test, *p* = 0.036).
3. As a result of the open exposure technique, the presence of gingival damage was connected with the success of canine eruption (chi-square test, *p* = 0.030).

The statistical analysis did not reveal significant differences between cases treated via buccal versus palatal exposure, nor between maxillary and mandibular cases. Due to the limited sample size in these subgroups, they were combined into a single analytical category. Nonetheless, potential differences between these groups cannot be excluded, and larger sample sizes will be required for more robust evaluation.

## 4. Discussion

The literature provides valuable insights into the causes of failed impacted canine exposure procedures; however, evidence specifically addressing surgical factors remains limited [12,13,14,15,16,17,18,19,20]. Becker et al. analyzed 28 cases of failed exposures and emphasized the critical importance of accurately diagnosing the position of the impacted canine and developing an appropriate case-specific treatment plan guided by effective orthodontic mechanics [11]. Failures involving both surgical and orthodontic components were attributed to factors such as improper planning of the exposure technique, suboptimal timing of the surgical intervention, excessive orthodontic traction, and the potential development of ankylosis secondary to surgical or orthodontic procedures [11].

Grisar et al. [12] analyzed 153 impacted maxillary canines in a retrospective study and found that the rate of impacted canine exposure failure was 4%. Vertical height and angulation affect aesthetic outcomes, and increased age is linked to a higher rate of failure when exposing impacted canines. Kim et al. [20] concluded that, in order to increase the success rate of forced eruptions of impacted canines, age and apex formation status should be taken into consideration to determine the length of treatment.

In the present study, 214 cases of impacted canine exposure were analyzed. In total, 191 successfully erupted, 23 failed to erupt after a first surgical procedure, and 8 failed to erupt after a second surgical procedure. The total failure rate in the present study is similar to that reported in the literature (3.7%), and the total complication rate was 7%.

The failure and complications analysis was divided into orthodontic and surgical procedures. Most of the cases were related to orthodontic factors (43.5%), as compared to surgical factors (8.6%). This disparity may be attributed to the nature of orthodontic treatment, which is typically more prolonged and involves multiple sequential procedures, whereas a surgical intervention is generally limited to a single event. It is also important to recognize that orthodontic considerations can influence the choice of surgical approach and may contribute to the overall treatment outcome.

A significant challenge arises when an obstruction—such as close proximity to the lateral incisor root—limits the feasible direction of orthodontic traction. In these situations, the surgeon may be compelled to select a suboptimal surgical approach to allow the orthodontist to guide the canine into the arch while avoiding damage to adjacent structures. However, such compromises in surgical access or vector control may increase the risk of treatment failure [21].

The surgical procedure itself may serve as a risk factor influencing canine eruption. Our findings indicate that open exposure is associated with a higher eruption success rate compared with closed exposure, likely due to the creation of an optimal exposure surface. This requires adequate removal of soft and hard tissue and effective bleeding control, allowing the orthodontist to securely bond an attachment to the canine and apply controlled traction. However, open exposure is not always feasible in patients with a high smile line, as it may result in gingival damage and compromised esthetic outcomes [10,22,23]. Surgeons must therefore carefully assess and manage these anatomical limitations to optimize treatment results.

The present study identified several risk factors influencing treatment outcomes.

**Figure 3:**
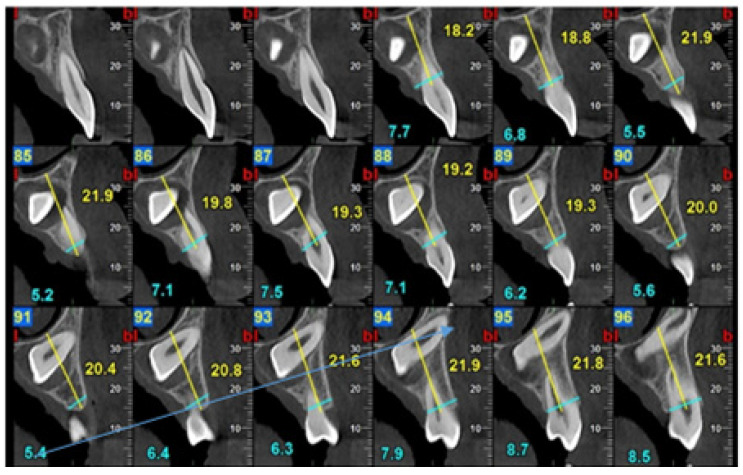
CBCT of impacted canine and root apex location in the buccal cortical plate (blue arrow).

Factors related to an impacted tooth: centrally positioned canine crowns within the alveolar ridge were associated with higher rates of failure and complications. This position poses considerable surgical and orthodontic challenges, often requiring the extensive removal of buccal or palatal bone to gain adequate access to the crown. Such procedures increase the risk of bleeding, limit visibility, and complicate surgical execution. A meticulous technique is therefore essential to avoid gingival injury, control bleeding, and maintain a dry field to ensure reliable bonding of the orthodontic attachment. The analysis of failure cases further demonstrated a correlation between canine apex proximity to a cortical plate (buccal or palatal) and an increased likelihood of eruption failure (Figure 3).

Cortical bone differs significantly from trabecular bone due to its denser, more compact, and less cellular structure. Bone density plays a critical role in tooth movement, as orthodontic tooth movement occurs more readily in less dense bone. Therefore, it is reasonable to suggest that the movement of teeth with roots located in the cortical plate presents a greater challenge. However, our findings on this issue are limited, and there is sparse literature available on this topic. Further research is necessary to better understand this correlation and its implications for treatment planning and outcomes.

The timing of surgical exposure for impacted canines is crucial. Our findings indicate that surgeries performed shortly after diagnosis had a significantly higher failure rate. While the current literature does not strongly support this observation, it is plausible that insufficient orthodontic preparation plays a role. Factors such as inadequate space opening for the canine, insufficient jaw expansion, or failure to reposition neighboring teeth that block the canine’s eruption path could contribute to these failures. Therefore, it is essential to ensure adequate space and thorough pre-surgical preparation to enhance the likelihood of successful canine eruption.

Lateral incisor root resorption caused by impacted canines was found to have a significant relationship with the failure of impacted canine exposure. This resorption results from direct contact between the impacted canine and the incisor root. The proximity between these teeth indicates clinical complexity and introduces significant challenges during the exposure procedure [24,25]. In the present study, the logistic regression analysis demonstrated that the odds of treatment failure were significantly higher in cases involving root resorption (OR = 4, *p* = 0.025). Exposure type has a substantial impact on eruption probability, suggesting that root resorption of adjacent teeth may hinder the eruption process. This finding highlights the critical need to address root resorption as a key factor in improving treatment outcomes. In such cases, it is not an option to forego exposing the impacted canine, as the primary goal of orthodontic treatment is to prevent and mitigate further damage to adjacent teeth. While orthodontic–surgical treatment remains the recommended approach, it is crucial to provide comprehensive informed consent to the patient and their family, outlining the complexities of the case and the heightened risk of failure. Clear communication ensures that all parties are aware of the potential challenges and can make informed decisions regarding treatment.

### 4.1. Associated Pathologies

Pathologies associated with canine impaction, identifiable through radiological imaging, may represent a risk factor for treatment failure. However, in the present study, the number of cases presenting such pathologies was limited, and further research is required to clarify potential associations.

In this cohort, the most common pathology associated with impacted canines was odontoma, observed in 3.2% of cases; six of the seven affected canines successfully erupted following exposure. A dentigerous cyst was identified in one case (0.4%), and this canine ultimately failed to erupt. Zhang et al. analyzed 2082 cases of dentigerous cysts and reported that approximately 5% were associated with impacted maxillary canines [26]. In their study, some canines erupted spontaneously after marsupialization, whereas others remained impacted. These findings support the hypothesis that dentigerous cysts may act as a preoperative risk factor, creating an additional barrier to successful orthodontic traction. Larger studies are needed to better define the impact of these pathologies on treatment outcomes.

### 4.2. Risk Factors Related to Intraoperative Factors

Surgical approach during the operation for exposure: A significant association was identified between the surgical technique and eruption success. Open exposure demonstrated a higher likelihood of successful eruption compared with closed exposure (B = 2.452, *p* = 0.018), with an odds ratio of 11.616. This finding is consistent with clinical reviews reporting technique-specific differences in periodontal morbidity and eruption outcomes [13,27,28,29]. The open exposure technique in the present study was correlated to gingival damage but also to the success of canine eruption. The literature suggests that closed exposure may limit the orthodontist’s ability to apply sufficient force to overcome early ankylosis, potentially contributing to treatment failure. In contrast, open exposure provides direct access to the crown, enabling the application of adequate orthodontic traction and thereby reducing the risk of ankylosis-related failure [29].

In addition to ankylosis, closed exposure may contribute to the formation of scar tissue, which can obstruct the traction chain or compromise bracket bonding, potentially necessitating a second surgical exposure [23]. These drawbacks should be carefully considered when selecting the most appropriate exposure technique.

Ankylosis itself may arise from several mechanisms. Closed exposure can limit the application of adequate orthodontic force, increasing the risk of ankylosis. Excessive masticatory pressure or trauma causing localized injury to the periodontal membrane may also initiate ankylosis. Such injuries can trigger ossification during healing and create localized metabolic disturbances around the tooth. Additional contributing factors include thermal trauma from low-speed burs during exposure, chemical trauma from 35% phosphoric acid, and mechanical trauma to the cervical periodontal ligament resulting from inappropriate direction or magnitude of orthodontic forces. Consequently, ankylosis may be induced by surgical or orthodontic interventions, but it may also occur spontaneously, as reported by Becker et al. [11]. Emerging evidence highlights the importance of orthodontic mechanics, appliance stability, and close interdisciplinary coordination in optimizing outcomes for impacted canine treatment [27,28,29].

The present study achieved 14% success for eruption of ankylosed canines following second operation and luxation. This finding indicates that luxation may represent an effective therapeutic strategy for managing tooth ankyloses by disrupting the bony bridge, thereby enabling the tooth to erupt [11]. This approach is supported by the study conducted by Becker et al. [9] in which three out of seven cases achieved successful eruption after luxation was performed [11]. When complications occur and a tooth fails to erupt after the initial attempt, a careful reevaluation of the case is critical before deciding on extraction. Reoperation may be considered following orthodontic consultation. If the tooth is ankylosed, has a poor prognosis, or poses a risk to adjacent structures, extraction may be the most viable option. It is important to note, however, that opinions differ regarding the use of luxation for managing impacted canines. Some proponents advocate for gentle tooth movement within the bone to loosen attachments and diagnose potential ankyloses. However, others caution that the procedure itself may induce ankylosis due to the associated bleeding and inflammatory response [29].

Given the limited data on luxation in the present study, further research is recommended to validate the hypothesis and clarify the risks and benefits of this approach.

### 4.3. Other Risk Factors for Complications

#### Gingival Damage

A connection was found between the open exposure technique and gingival damage. This was correlated with the success of canine eruption. The open exposure approach, associated with a higher success rate, was found to have a high correlation with gingival damage (*p* = 0.036).

The literature lacks sufficient information on this topic, but it can be hypothesized that damage to the soft tissue results from strong orthodontic forces and aggressive surgical exposure procedures. Sometimes, this cannot be avoided when pulling the canine into the dental arch. Therefore, we suggest informing patients that a side effect of impacted canine exposure may include damage to the soft tissue, scarring, recession, gingivitis, and other gingival reactions.

**Figure 4:**
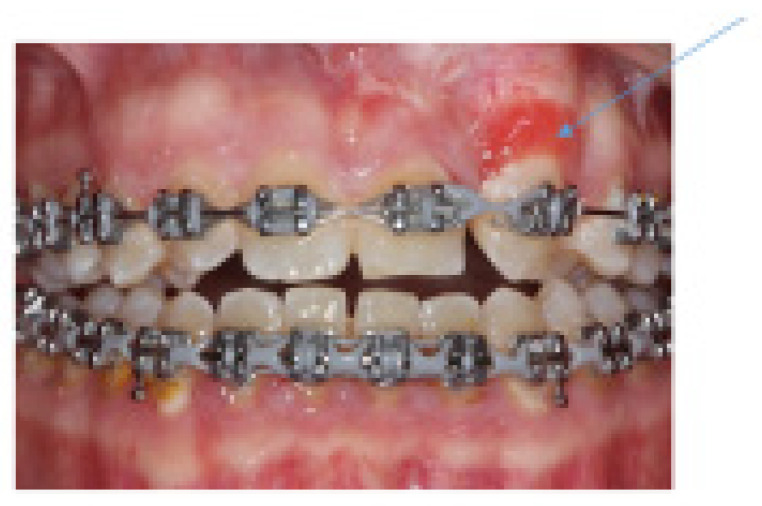
Juvenile spongiotic gingival hyperplasia (blue arrow).

The findings highlight treatment complications. Surgical treatments can lead to gingival damage, such as scarring and gingival recession. Based on our retrospective clinical observations, these complications arise when there are procedural faults, for instance, if the operation becomes too extensive or aggressive. Additionally, operating on keratinized tissue of insufficient thickness (below 3 mm) can pose a risk of damage to the soft tissue [12]. Surgical irritation of the flap might result in a gingival reactive response, such as juvenile spongiotic gingival reactive reactions (Figure 4).

Vitality of tooth: The evidence regarding vitality loss following surgical exposure remains inconclusive, and such damage may result from either surgical trauma or orthodontic forces. Further investigation is needed to clarify the underlying mechanisms and to develop strategies for preventing this complication.

### 4.4. Study Limitations

This study has several limitations. First, its retrospective, single-center design restricts the diversity of treatment practices, patient demographics, and case-management approaches, which may limit the generalizability of the findings. Second, the relatively small number of failure cases reduces the statistical power for subgroup analyses and increases the risk of over-interpreting observed associations. Larger, multicenter prospective studies are needed to validate these findings and provide a more comprehensive understanding of the factors influencing treatment outcomes.

### 4.5. Study Generalizability

The study population’s characteristics may differ from those of other regions or institutions, particularly with respect to treatment protocols, patient demographics, and underlying anatomical or pathological variations. As a result, the findings are most applicable to centers that employ similar surgical and orthodontic approaches. Variability in clinical techniques (e.g., open vs. closed exposure), practitioner expertise, and available resources may further limit the generalizability of the results.

Despite these limitations, the study offers valuable insights into the management of impacted canines, particularly regarding preoperative risk factors and the dynamic interaction between surgical and orthodontic components. These findings may contribute to the refinement of broader treatment guidelines and support more individualized, evidence-based decision making in clinical practice.

## 5. Conclusions

1. A study of 214 cases at the university revealed a failure rate of 3.7% for impacted canines’ exposure. This closely reflects the reported rate in the literature when including cases of re-exposure that eventually succeeded.
2. Surgical factors have a lower risk of failures and complications compared to orthodontic factors: 8.6% versus 43.5%, respectively. Therefore, special attention and close follow-up are essential to identify and promptly treat developing complications. This is especially important in cases where the root apex is located in one of the cortical plates, the impacted tooth crown is in the middle of the alveolar ridge, or there is root resorption or pathology.
3. The risk factors are as follows:
  - Impacted canines positioned centrally within the alveolar ridge, particularly when the root lies in close proximity to a cortical plate, show a significantly increased risk of eruption failure due to anatomical constraints (*p* = 0.025).
  - Root resorption of the adjacent lateral incisor is a strong predictor of poor outcomes, increasing the odds of treatment failure approximately fourfold (*p* = 0.025).
  - Timing of the exposure surgery is critical; performing the procedure shortly after diagnosis is associated with a significantly higher risk of failure (*p* = 0.006).
4. Open exposure proved significantly more successful than closed exposure; the odds for success are 11.12 (*p* = 0.009).
5. Gingival damage was associated with success (*p* = 0.03) and with open exposure (*p* = 0.036).

### Recommendations

- Surgeons and orthodontists should pay special attention to impacted canine location, especially crown locations in the middle of the ridge and root locations near one of the cortical plates.
- Patients should be informed about the possibility of failure in cases where root resorption of the lateral incisor is present.
- It is important to plan the exposure timing carefully.
- Whenever possible, open exposure should be considered to lower the rate of reoperation.
- Damage to the gingiva should receive more attention, especially if the exposure is performed in an aesthetic location (buccal aspect).

## Figures and Tables

**Table 1:** Analysis of 214 cases.

Description	No. of Cases	Reason	Failure	Complication
Canine did not erupt after 1st attempt	23	Orthodontic 10 cases Surgical: 3 cases Combination 9 cases	8 canines were extracted	
Canine erupted after the 1st attempt	191	Orthodontic 10 cases: Surgical: 2 cases Combined: 6 cases		18 cases

**Table 2:** Analysis of Radiographic and Clinical data.

		Number of Teeth	Percent
Impacted canine tooth number	13	92	43.0
	23	100	46.7
	33	10	4.7
	43	12	5.6
Location of the impacted canine	Buccal	79	36.9
	Palatal\Lingual	127	59.3
	In the middle of the alveolar ridge	8	3.7
Yamamoto classification	I	70	32.7
	II	117	54.7
	III	4	1.9
	IV	21	9.8
	V	2	0.9
Root resorption of the proximal tooth	No	196	91.6
	yes	18	8.4
Associated pathology	No	206	95.8
	Yes	8	3.7
Pathology type	Dentigerous cyst	1	0.4
	odontoma	7	3.2

**Table 3:** Relationship between impacted canine crown position and treatment outcome.

Crown Location of Impacted Canine	Failure, No (%)	Success, No (%)	Total, No
Buccal	10 (12.7)	69 (87.3)	79
Palatal/lingual	10 (7.9)	117 (92.1)	127
Central	3 (37.5)	5 (62.5)	8
Total number	23	191	214

**Table 4:** Analysis of 18 cases of complications.

Sort of Complication	No of Cases	Reason
Scar in the soft tissue	8	2—surgical 2—orthodontic 4—combined
Loss of vitality	2	orthodontic
Root resorption	4	orthodontic
Tooth mobility	2	orthodontic
Gum recession	2	combined

## Data Availability

The datasets generated during and/or analyzed during the current study are available from the corresponding author on reasonable request.
